# Value of Diagnosis in Filling Teeth

**Published:** 1908-08-15

**Authors:** J. V. Couzett


### VALUE OF DIAGNOSIS IN FILLING TEETH.

BY J. V. COUZETT, D.D.S.

In making any filling or attempting any operation or restoration upon the teeth we must first make a careful study of the conditions as they present themselves. Every case that comes to us has some point or points of difference and every case must be studied by itself. We must make a general examination of the whole mouth to determine the susceptibility of the patient, the condition of the fluids of the mouth and the condition of the mucous membrane, for upon these conditions must we depend for a knowledge of how great must be the extension of our cavity for the prevention of recurrence of decay. It will not be necessary, for example, to carry the margins of our cavities quite so far into immune territory in cases that present where there is quite a general immunity and in which the secretions of the mouth are normal and the mucous membranes are in a state of good health, while in cases of extreme susceptibility it is absolutely necessary to give the widest extension possible.

The occlusion must be carefully studied and any abnormalities observed. Particular attention should be paid to the facets worn upon the occlusal or incisal surfaces of the teeth, as these are sure indications of the direction and stress of masticatory movements. If a filling is made without observing these things the operator is liable to get into trouble by reason of the fact that a power is brought into action upon his filling with which he has not reckoned, and consequently has made no provision to meet, and his filling will fail in consequence.

I once made a filling in the mesioincisal surface of an upper right lateral incisor and was very proud of what I thought was a very beautiful filling, but what was my chagrin to have the patient return in a short time with the incisal portion of my filling ground through and the whole filling loose and a complete failure. I now, tardily, made an examination of the occlusion and found that the sharp point of the right lower cuspid in its excursions in mastication trailed over the incisal surface of the upper lateral in such a way that it wore a groove in the incisal surface, which I had not noticed by reason of the decay of the tooth. I remade the filling, ground off the point of the offending cuspid and had no further trouble.

In the same way we sometimes find long, sharp cusps upon bicuspids that fit into deep sulci of the occluding bicuspid. In restoring these it is well to relieve the occlusion by grinding off a portion of the antagonizing bicuspid. Not only must we observe the normal occlusion and the natural excursions of the teeth during mastication, but we must carefully look for signs of abnormal excursions of the teeth during sleep, as in the habit of grinding the teeth at night, or as the result of nervous habit during periods of excitement. I made a filling for a lady in the incisal surface of an upper right cuspid and it was ruined by the habit of grinding her teeth at night. It did not seem possible that she could reach that particular place with any of her lower teeth, but after trying for some time I found that a cusp of one of the lower teeth did fit into it during a specially peculiar motion of the jaw. We fixed that cusp so it would not do the mischief again, and another lesson in studying occlusion was learned. There are some cases, however, that seem to defy all of our precautions in this respect. One case comes to my mind, that of a lady with a highly organized nervous temperament, who presented with the lingual and incisal surfaces of her teeth very badly ground down. She asked for a porcelain restoration, which, of course, would have been the height of folly, for porcelain would not have stood the stress of that tremendous strain for fifteen minutes after she got into action some night. During the daytime and upon our examinations I had never been able to make her close her teeth in such a manner as to reach those fillings, but she presented herself after a time with a groove running mesiodistally clear across those fillings and it was always done at night. In this particular case I built the lingual surfaces and over the incisal surfaces of both central incisors with platinized gold, and although a deep groove has been worn in both fillings they are still doing good service.

Not only must the direction and peculiarities of the occlusion be observed, but the strength thereof must be carefully noted. For if we place a filling in a tooth upon which there is great stress and do not anchor the filling sufficiently to resist the force that is brought to bear upon it, the filling will fail in time either by being bodily crushed out of the cavity or by having its form so changed by the flow of gold under stress that we shall have a recurrence of decay. In many of these cases it will be noticed that there has been a wearing of the gold upon some portion of its occlusal surface, and that surface not being heavy enough has not only been worn under the stress, but there has been a "flowing" of the entire mass of gold in the direction of the stress, consequently there has been a slight change in the shape of the filling and a recurrence of decay due to leakage. The remedy is to note the direction and strength of the stress of mastication and then at those points that must resist heavy occlusion cut wide and deep that you may have a large mass of gold to resist the stress at that point. Again we must note in what portion of the tooth our cavity is located, as greater strain comes upon certain portions of the tooth than others. For instance, we know that the mandible in mastication normally closes inside of the superior maxillary. The lower teeth closing inside of the upper teeth, therefore, the lower jaw exerts a force toward the mesial surfaces of the teeth. The stress therefore comes upon the mesial surfaces of the upper teeth and upon the distal surfaces of the lower teeth. In making our filling, then, it behooves us to anchor more firmly the filling in the mesial surfaces of the upper teeth and in the distal surfaces of the lower teeth than in the opposite surfaces of the same teeth. We should also note the masticatory habit in the linguo-buccal occlusion, for in nearly all cases, by closely observing the facets worn upon the surfaces of the teeth, we will find that each individual commonly exerts more stress in one direction than in the other. That is, he may commonly chew his food with a movement from right to left, or from left to right. In many cases it is important to know which way the greater stress is liable to come, that a buccal or, it may be, a lingual wall may be protected from a stress that may be too great for it to bear. I find that it is usually the lingual cusps of the right upper and the left lower teeth and the buccal cusps of the left upper and right lower teeth that suffer the most, indicating that the majority of cases that have come under my observation habitually grind their food with from right to left movement. This may be but a coincidence, but it is an interesting one and one that will bear further observation. But in every case involving any considerable portion of the occlusal surfaces of bicuspids and molars it is wise to carefully note this particular form of stress, and then protect any weak cusp that may have to bear the brunt of that stress. Failure to do so will invite the destruction of a cusp, an accident that is peculiarly unfortunate, as it nearly always means the loss of the crown of the tooth.

Again it is our duty to note the condition of the pericemental membrane, for upon its condition must depend, in a large degree, our choice of filling material. We know, or should know, that it requires a considerable force to properly condense gold foil, and if we attempt a large gold restoration in a tooth that is decidedly "lame" as a result of a pyorrhea, or some form of pericemental irritation, or in a tooth that has no occlusion, and as a result has a "soft", tender membrane, we will find before we go very far in the operation that the patient is suffering intensely under the strain of malleting, and if we persist we will probably have a very sore tooth if we have not permanently disabled it. In such teeth a large gold filling is decidedly contraindicated. To the average man all this may sound rather nonsensical, as he will think that all of these observations will take more time than it would take to make a good-sized filling. On the contrary, it will help you make your fillings more quickly and surely more perfectly. At first it will take time, I admit, but by continued practice and observation all of these points will be noticed in a glance and far quicker than it takes to tell. Then with the conditions in mind, knowing the corrections that are to be made, there immediately arises in the mind's eye a picture of the desired result, and any man that commences any work, be it the plan of some great cathedral or skyscraper office building, a picture or a statue, my lady's bonnet, or your latest suit of clothes, or, forsooth, an operation upon any particular tooth, and has not in mind a perfect picture of the finished product, is going either to make a failure or is going to spend far too much time in its accomplishment. Then if you have it not, first form the habit, and do it now, of knowing what your are going to do before you do it, know how you are going to do it, and know why. If you do this you see every step of the procedure mapped out before you, you know just the instrument that you need to accomplish each step, and quickly and surely each step succeeds the other until in time that is a surprise to you and a pleasure to your patient, the operation is completed.—*Dental Digest.*

